# Perception of health-related quality of life in children with chronic kidney disease by the patients and their caregivers: Multicentre national study results

**DOI:** 10.1007/s11136-013-0416-7

**Published:** 2013-04-18

**Authors:** Katarzyna Kiliś-Pstrusińska, Anna Medyńska, Irena Bałasz Chmielewska, Ryszard Grenda, Agnieszka Kluska-Jóźwiak, Beata Leszczyńska, Julita Niedomagała, Ilona Olszak-Szot, Monika Miklaszewska, Maria Szczepańska, Marcin Tkaczyk, Agnieszka Urzykowska, Anna Wasilewska, Katarzyna Zachwieja, Maria Zajączkowska, Helena Ziółkowska, Ilona Zagożdżon, Danuta Zwolińska

**Affiliations:** 1 Department of Paediatric Nephrology, Wroclaw Medical University, ul. Borowska 213, 50-556 Wrocław, Poland; 2 Department of Paediatric and Adolescent Nephrology and Hypertension, Medical University of Gdansk, Gdansk, Poland; 3 Department of Nephrology, Kidney Transplantation and Hypertension, The Children’s Memorial Health Institute, Warsaw, Poland; 4 Department of Paediatric Cardiology and Nephrology, Poznan University of Medical Sciences, Poznan, Poland; 5 Department of Pediatrics and Nephrology, Medical University of Warsaw, Warsaw, Poland; 6 Nephrology Division, Polish Mother’s Memorial Hospital Research Institute, Łodź, Poland; 7 Department of Nephrology, Children Hospital, Toruń, Poland; 8 Polish-American Children’s Hospital, Jagiellonian University, Krakow, Poland; 9 Clinic of Paediatrics, Nephrology and Endocrinology, Silesian Medical University, Zabrze, Poland; 10 Department of Pediatrics and Nephrology, Medical University of Białystok, Białystok, Poland; 11 Department of Pediatric Nephrology, Medical University of Lublin, Lublin, Poland

**Keywords:** Chronic kidney disease, Children, Family life, Health-related quality of life

## Abstract

**Objective:**

The aim of the study was to analyse the health-related quality of life (HRQoL) in Polish children with chronic kidney disease (CKD) dependant on the CKD stage, treatment modality and selected social life elements in families of the patients. Furthermore, potential differences between self-report and parent/proxy reports and the factors influencing them were assessed.

**Methods:**

A total of 203 CKD children (on haemodialysis (HD), peritoneal dialysis (PD) and conservative treatment (CT)) and their 388 parent/proxies were enrolled into a cross-sectional national study. The demographic and social data were evaluated. We used the Paediatric Quality of Life Inventory 4.0 Generic Core Scales to assess the HRQoL in children.

**Results:**

Health-related quality of life scores for all CKD groups were significantly lower in all domains compared with population norms, the lowest one being in the HD group. In CT children, HRQoL did not depend on the CKD stage. Both parents assessed the HRQoL of their children differently depending on their involvement in the care. There are differences between the HRQoL scores of the children and their parents.

**Conclusion:**

The HRQoL in children with CKD is lower than in healthy children. This is already observed in the early stages of the disease. The disease itself influences the child’s mental state. Children on HD require special support on account of the lowest demonstrated overall HRQoL. Children’s lower rating of the quality of life observed by their parents may render the patients unmotivated and adversely affect their adjustment to life in later years. It may also create conflicts between the parents and the children.

## Introduction

Chronic kidney disease (CKD) burdens the patients biologically, socially and psychologically [1–3]. It affects the patients’ quality of life (QoL). Health-related quality of life (HRQoL) is a sub-domain of QoL and can be defined as the subjective perception of how health-related factors impact the well-being and life satisfaction [4]. It is increasingly recognized as an important outcome of the CKD treatment [5–7]. This applies not only to the patient’s current life situation but it may also influence the patient’s functioning in the future. It is particularly important among paediatric patients due to the extended survival time among children with CKD who, having reached adulthood, will find themselves in a different social and economic reality [8–10].

The use of HRQoL measurements related to CKD children is a relatively new field of research. The results in the published studies are inconclusive and pertain to small groups of patients at different CKD stages [11–13]. Eijsermans et al. showed that the perceived QoL among children on dialysis and the general population did not differ [14]. On the other hand, research by Gerson et al. [15] showed that compared with control subjects, patients with CKD (treated conservatively and undergoing renal replacement therapy) had lower overall satisfaction regarding their health. McKenna et al. [16] concluded lower HRQoL in children treated conservatively (*n* = 20), dialysis (*n* = 17) and kidney transplant (KT, *n* = 26) in comparison with healthy children. However, no statistically significant differences were reported based on the treatment methods for CKD. In turn, Goldstein et al. [11] examined children with end-stage renal disease (ESRD) on peritoneal dialysis (PD), haemodialysis and after KT. He demonstrated that ESRD patients’ HRQoL scores were significantly lower than healthy controls but concluded that there was no difference between HD and PD patients.

Various tools were used to measure QoL, thus making the results difficult to compare [17]. Moreover, in most of the studies involving the families of CKD patients, either the children or their parents/guardians were examined without taking into account their interrelationships [18]. So far, the psychosocial situation of children with CKD or their quality of life has not been analysed in Poland.

Therefore, the main goal of the present study was to analyse HRQoL in Polish children with CKD depending on the stage and treatment for CKD, and some life situation elements in their families. Furthermore, potential differences between patient- and parent/proxy reports and the factors influencing them were assessed. It was anticipated that the study would help to understand the impact of CKD on the functional health status of children and to determine if, when and how to support CKD patients in order to improve their quality of life.

The Paediatric Quality of Life Inventory (PedsQL) 4.0 was used to assess HRQoL. It has been demonstrated to be reliable and valid across a wide spectrum of chronic childhood diseases in a number of populations from different countries [19, 20].

## Methods

### Participants

Eleven out of 12 paediatric nephrology centres in Poland offering care for children with CKD participated in a cross-sectional study. The study was conducted among patients and their parent/proxies between September and December 2011. The study protocol adhered to the Declaration of Helsinki and was approved by the Ethics Committee of Wroclaw Medical University. Written informed consent was obtained from all children over 16 years and all the parents. Verbal assent was obtained from patients under 16 years of age when possible.

Inclusion criteria for children are as follows: (1) over 2 years of age, (2) stage II of CKD or higher according to KDOQI [21] based on the estimated glomerular filtration rate (eGFR) by the Schwartz formula [22], (3) CKD diagnosed at least 3 months prior to the begin of the study and (4) informed consent. Exclusion criteria for children are as follows: (1) history of severe to profound mental retardation, (2) renal, other solid-organ, bone marrow or stem cell transplantation, (3) cancer/leukaemia diagnosis, (4) hospitalization within 14 days (with the exception of a hospital stay for HD or PD), (5) initiation or change of dialysis modalities within the past 30 days and (6) a significant life event unrelated to their kidney disease in the past 30 days, such as losing a family member.

The patients were divided into 3 groups based on the treatment modality: conservative treatment (CT), haemodialysis (HD) or peritoneal dialysis (PD).

### Methods

The study was performed in two steps. Step I. A medical chart review was performed to obtain the following information: primary diagnosis of kidney disease, patient’s age at CKD diagnosis, duration of the illness, time of nephrology care, additional non-renal comorbidities, place of living and the distance from a nephrology centre and family history. Additionally, school-age children and their parents were interviewed about schooling level and special education requirements (supplementary tutoring or an individualized education programme). Analysed data for parents were age, education, job type, care of the sick child (main caregiver/“second” parent) as well as the family structure: parents/single parent, siblings/only child. “Second parent”—we used this term to describe the parent who spends less time taking care of a sick child.

Step II. To assess the HRQoL in children, we used the PedsQL 4.0 Generic Core Scales [19, 20, 23]. We received the user agreement from Christelle Berne, Mapy Research Institute in Lyon, France. The tests were designed according to the general protocol and administration guidelines. This measure comprises child report (5–18 years of age) and parent report (2–18 years of age) of the child’s HRQoL. The 23-item version of PedsQL was used for children 5–18 years of age and for their parents. The 21-item version was used only for the parents of children 2–4 years of age. The PedsQL assesses physical (8 items), emotional (5 items), social (5 items) and school/nursery school (5/3 items) functioning in adolescents and children. For children 8–18 years of age and parent/proxy report formats, items are rated on a 5-point ordinal scale to indicate the problems children encounter in various areas of functioning, ranging from 0 (never) to 4 (almost always). For younger children, the ordinal scale is reworded and simplified to a 3-point scale: 0 (not at all a problem), 2 (sometimes a problem) and 4 (a lot of problem). From the sum of the raw scores from the 23 (or 21) items, a summary score ranging from 0 to 100 can be calculated, with higher scores indicating higher HRQoL. The results obtained by main caregivers and the “second” parents were analysed separately.

### Statistical analyses

Means, standard deviations, medians, quartiles, frequencies and percentages were reported to describe the data as appropriate. Quantitative variables were tested for normality of the distribution using the Kolmogorov–Smirnov test. The Mann–Whitney test, the Student’s *t* test and the Kruskal–Wallis test were used, to compare the different studied groups. The means of PedsQL from the three groups (CT, PD and HD) were compared with a sample of healthy children reported by Varni et al. [19] using Student’s *t* test. Qualitative variables were presented as numbers and percentages. The chi-squared test or Fisher’s exact test was used for comparison between groups. Univariate analyses were carried out in order to determine what independent variables were associated with children`s scores. Next, multivariate analysis was conducted to evaluate the relationship between the PedsQL domain scores and variables that were found to be associated in the univariate analyses or supported by practice observations. Statistical analyses were performed using R for Windows, version 2.15.1 (The R Foundation for Statistical Computing, Vienna, Austria) and MedCalc for Windows, version 12.3.1.0 (MedCalc Software, Mariakerke, Belgium). A *p* value of <0.05 was considered statistically significant.

## Results

A total of 203 children with CKD were enrolled into this study. Data from 388 of their parent/proxies (196 women and 192 men) were taken into consideration. The characteristics of patients and their parent/proxies are provided in Tables 1, 2. About 30 % of school children were tutored and the remaining children received universal education. Among the PD children, the patients were tutored at home in 55.2 % of the cases, versus 66.7 % of HD patients, versus 17.4 % of patients treated conservatively. A statistically significant difference in learning methods was demonstrated only between CT patients and children on dialysis (*p* = 0.01).

**Table 1:** Basic characteristics of CKD children

Parameter	*n*	%
Age, mean ± SD (years)	10.95 ± 4.95	
*Gender*		
Female	80	39
Male	123	61
*Cause of CKD*		
Chronic glomerulonephritis	32	15.8
Anomaly of kidney and urinary tract	102	50.3
Hereditary kidney disease	34	16.7
Others	23	11.3
Unknown cause	12	5.9
*Comorbidity*		
Yes	34	16.8
No	169	83.2
*Family renal history*		
Yes	27	13.3
No	176	87.7
*Place of residence (size)*		
Village and town <50, 000 inhabitants	88	43.4
Town 50, 000–100, 000 inhabitants	51	25.1
Town >100, 000 inhabitants	64	31.5
Age of CKD diagnosis, median (quartiles) (years)	2.02 (0.17; 8.0)	
CKD duration, mean ± SD (years)	6.91 ± 4.8	
Duration of nephrology care (years)	6.41 ± 4.52	
*CKD treatment modality*		
Conservative treatment	137	67.5
CKD stage 2	30	22
CKD stage 3	58	42
CKD stage 4	40	29
CKD stage 5	9	7
Haemodialysis	25	12.3
Peritoneal dialysis	41	20.2

*CKD* chronic kidney disease

**Table 2:** Basic characteristics of parents and family of CKD children

Parameter	*n*	%
Mothers	196	100
Age, mean ± SD (years)	37.97 ± 6.74	
*Mother`s education level*		
Elementary/trade school	87	44.4
High school	72	36.7
University degree	37	18.9
*Employment*		
Yes	129	61.2
No	67	38.8
*Healthy*		
Yes	165	83.3
No	31	16.7
Fathers	192	100
Age (mean ± SD) (years)	40.91 ± 7.39	
*Father`s education level*		
Elementary/trade school	124	64.6
High school	42	21.9
University degree	26	13.5
*Employment*		
Yes	165	86
No	27	14
*Healthy*		
Yes	164	85.4
No	28	14.6
*Family* ^*a*^		
Full	175	86.2
Single-parent family	25	13.8
*Main caregiver* ^*a*^		
Mother	175	87.5
Father	19	9.5
Both parents	6	3.0
*Sibling*		
Yes	163	80.3
No	40	19.7

No data were available on 7 mothers (4 are deceased, and in the case of 3 children, living in social welfare centres—no contact) and 11 fathers (2 are deceased, and in 3 cases, children live in social welfare centres, no contact is available in the remaining cases)

^a^Three children live in a social welfare centre

The PedsQL test results are presented in tables and include the size of the studied groups. The differences in group size stem from the properties of the PedsQL test (child report for children aged 5–18, parent/proxies report for children 2–18 years of age). Results of missing or incomplete tests were discarded.

HLQoL scores for all CKD groups were significantly lower in all domains compared with international population norms for healthy children (*p* < 0.0001). We are aware the best effects would bring comparing the Polish CKD children results to Polish norms. However, there are no such norms for Polish or even European children. Hence, there is a need of very cautions interpretation of the results. It should be stated that the norms conducted by Varni et al. [19] are based on a large number of studied subjects, which allows to conclude that the influence of socio-demographic factors was notable reduced.

Statistically significant differences were also indicated between groups of children with CKD identified according to the method of treatment (Table 3-A). Patients treated with dialysis assessed their physical and social functioning lower than patients treated conservatively. No differences were found in this respect based on the type of dialysis. There were no differences between all groups of patients in their perception of emotional and school functioning. However, a tendency to lower assessment in the last domain was evident in the HD group when compared with the other children. The HD patients obtained significantly lower overall HRQoL scores than those treated conservatively or with PD. CT children did not differ significantly in terms of the overall HRQoL, nor did they differ in the results of the individual subscales, dependent on the stage of CKD (Table 3-A).

**Table 3:** PedsQL 4.0 generic core scales from: (A). CKD child self-report, (B). parent/proxy reports (main caregivers), (C). “second” parent/proxy reports

	Physical functioning	Emotional functioning	Social functioning	School functioning	Overall HRQoL
(A). children, *n* = 171					
CT, *n* = 118	78.13 (63.28−7.5)	65.0 (55.0−80.0)	80.0 (60.0−90.5)	65.0 (50.0−75.0)	71.74 (58.15−80.98)
CKD 2–3, *n* = 75	78.13 (60.94−87.50)	65 (55−77.5)	80.0 (60−100)	65.0 (50−80)	69.02 (57.07−81.52)
CKD 4–5, *n* = 43	78.13 (65.63−84.38)	65.0 (55−80)	80.0 (65−93.75)	65.0 (55−70)	72.83 (60.87−79.08)
HD, *n* = 22	40.63 (18.75−65.63)	52.5 (35.0−75.0)	67.5 (50.0−80.0)	47.5 (35.0−65.0)	50.54 (40.22−64.13)
PD, *n* = 31	62.5 (50.0−75.0)	70.0 (56.25−73.75)	70.0 (50.0−90.0)	62.5 (50.0−75.0)	64.13 (51.09−75.0)
*p* value	**0.001** ^**ab**^	0.11	**0.02** ^**a**^	0.07	**0.001** ^**b**^
(B). Parents/main caregivers, *n* = 201					
CT, n = 136	75.00 (53.13−84.38)	65.0 (51.25−75.0)	75.0 (60.0−93.75)	60.0 (45.0−70.0)	66.3 (51.63−76.09)
CKD 2–3, *n* = 85	75.00 (50.0–87.5)	65.0 (55.0–80.0)	75.0 (60.0–95.0)	60.0 (45.0−70.0)	67.94 (51.09−78.26)
CKD 4–5, *n* = 51	71.88 (58.59–81.25)	60.0 (50.0–70.0)	70.0 (55.0–86.25)	55.0 (45.0–65.0)	65.22 (54.08–75.0)
HD, *n* = 24	51.56 (20.31–68.75)	50.0 (37.5–65.0)	62.5 (45.0–85.0)	40.0 (35.0–60.0)	49.46 (40.22–62.5)
PD, *n* = 41	56.24 (40.63–62.5)	55.0 (40.0–70.0)	55.0 (40.0–70.0)	55.0 (35.0–65.0)	54.35 (38.87–64.13)
*p* value	<**0.0001** ^**a**^	**0.006** ^**a**^	**0.0003** ^**a**^	**0.03** ^**c**^	**0.0001** ^**a,d**^
(C). “Second” parents *n* = 84					
CT, *n* = 61	75.00 (56.25–84.38)	60.0 (53.75–80.0)	80.0 (63.75–95.0)	65.0.0 (50.0–75.0)	66.67 (53.26–79.62)
CKD 2–3 *n* = 39	78.13 (56.26–84.38)	65.0 (60.0–80.0)	80.0 (70.0–95.0)	70.0 (50.0–75.0)	69.05 (59.5–80.44)
CKD 4–5, n = 22	71.88 (53.13–81.25)	55.0 (45.0–60.0)	72.5 (60.0–80.0)	60.0 (50.0–75.0)	65.76 (50.0–73.91)
HD + PD *n* = 23	53.13 (41.41–67.97)	55.0 (41.25–68.75)	65.0 (56.25–80.0)	55.0 (40–65)	56.53 (49.18–63.04)
*p* value	**0.006** ^**e**^	0.06^f^, **0.02** ^**h**^	0.09	0.08	**0.002** ^**g**^

Data were shown as median values and quartiles (first—third quartile). *CT* children on conservative treatment, *HD* children on haemodialysis, *PD* children on peritoneal dialysis

For children: ^a^CT versus PD and HD; ^b^ HD versus PD and CT. For parents/main caregivers: ^a^CT versus PD and HD; ^c^CT versus HD; ^d^ PD versus HD. For “second” parents: ^e^CT versus HD + PD; ^f^ CT versus HD + PD; ^g^CT versus HD + PD; ^h^CKD2–3 versus CKD4–5. Statistically significant differences are in bold (*p* < 0.05)

In the entire group of patients and in all subgroups determined by treatment methods, no significant relationship was noted between the individual components of the PedsQL test and CKD duration. No differences were indicated in the perception of the overall HRQoL and all its components based on age, age of CKD diagnosis, primary kidney disease, time of nephrology care, the size of place of residence, the distance from a nephrology centre, the type of education provided, parent`s education level and father`s employment. Male gender (in comparison with female gender) and mother’s employment were associated with higher physical functioning score (median value, quartiles: 75.0; 59.36–87.5 vs. 68.75; 40.63–82.03, *p* = 0.03; and 75.56; 60.94–87.5 vs. 68.75; 47.6–81.25, *p* = 0.02, respectively). Children from two-parent families reported better social functioning (68.0; 55.0–80.0) and observed higher overall QoL (68.93; 55.43–78.26) than children from single-parent families (63.0, 50.0–75.0, *p* = 0.02; 65.5, 51.0–79.34, *p* = 0.03, respectively). In a multivariate analysis, only connection between gender, mother’s employment and physical domain of PedsQL (coefficient 7.97, std. error 4.02, *p* = 0.05 and coefficient 8.85, std. error 4.04, *p* = 0.03, respectively) were found.

The main caregivers’ perception of the overall HRQoL and particular types of functioning in children with CKD was statistically significantly different dependent on CKD treatment methods (Table 3-B). The parents of CT children rated their quality of life much higher, including the individual subscales, in comparison with parents of children treated with HD as well as the parents of children treated with PD. The only exception formed the school functioning. It was determined that the parents of the HD children reported lower total scores not only when compared with the parents of children treated conservatively, but also with PD. Evaluations by the parents of the HD and PD children were not significantly different in partial subscales. The second parents of children treated conservatively rated physical functioning and overall HRQoL higher than the parents of dialysis patients. There were no differences in the other subscales (Table 3-C).

The main caregivers of CT patients did not differ in the perception of HRQoL of their children based on the stage of CKD (Table 3-B) as opposed to the “second parents” who rated emotional functioning in children at stages 2, 3 much higher than at stages 4, 5 (Table 3-C).

Overall, HRQoL and other subscale evaluation by children (the all CKD group) and their primary caregivers varied. In the CT group, the lower rating among the parents pertained to the overall HRQoL and its subscales with the exception of emotional functioning. In the HD group, no significant differences were demonstrated. In the PD group, the parents’ lower scores were observed in the overall HRQoL of the children, emotional functioning as well as physical and social functioning (Table 4).

**Table 4:** Comparison between PedsQL 4.0 generic core scales from child self-report and parent/proxy reports (main caregivers)

Characteristic	CT	HD	PD	Total
Parent/proxy	*N* = 118	*N* = 22	*N* = 31	*N* = 171
Child	*N* = 118	*N* = 22	*N* = 31	*N* = 171
*Physical functioning*				
Child	78.13 (63.28–87.5)	40.63 (18.75–65.63)	62.5 (50.0–75.0)	71.88 (53.13–84.38)
Parent/proxy	75.0 (52.34–84.38)	51.56 (21.88–68.75)	56.25 (43.75–63.28)	65.63 (46.88–81.25)
*p*	**0.05**	0.73	**0.05**	**0.05**
*Emotional functioning*				
Child	65.0 (55.0–80.0)	52.5 (35.0–75.0)	70.0 (58.75–71.25)	65 (55–80)
Parent/proxy	65.0 (50.0–75.0)	52.5 (40.0–65.0)	55.0 (40.0–70.0)	60 (45–70)
*p*	0.09	0.7	**0.04**	**0.009**
*Social functioning*				
Child	80.0 (60.0–95.0)	67.5 (50.0–80.0)	70.0 (50.0–90.0)	77.5 (60.0–95.0)
Parent/proxy	72.5 (55.0–90.0)	62.5 (45.0–90.0)	55.0 (40.0–70.0)	70.0 (50.0–90.0)
	**0.05**	0.82	**0.05**	**0.01**
*School functioning*				
Child	65.0 (50.0–75.0)	47.5 (35.0–65.0)	60.0 (50.0–75.0)	65.0 (46.25–75.0)
Parent/proxy	55.0 (45.0–70.0)	40.0 (35.0–60.0)	55.0 (35.0–65.0)	55. (40.0–65.0)
	**0.03**	0.2	0.13	**0.004**
*Overall HRQoL*				
Child	71.74 (57.61–81.52)	50.54 (40.22–64.13)	64.13 (53.26–75.35)	67.94 (54.35–78.3)
Parent/proxy	65.76(51.09–78.26)	49.46 (40.22–65.22)	55.44 (46.2–64.13)	60.87 (47.83–73.91)
	**0.03**	0.84	**0.03**	**0.008**

Data were shown as median values and quartiles (first—third quartile)

*CT* children on conservative treatment, *HD* children on haemodialysis, *PD* children on peritoneal dialysis

Statistically significant differences are in bold (*p* < 0.05)

## Discussion

The current study is the first to report data on HRQoL among children and adolescents with CKD in Poland. The material focused on children treated in almost all paediatric nephrology centres in the country; hence, the group under study can be considered as representative of CKD children among the Polish population. In the recent years, the HRQoL has been declared one of the most important goals of patient care besides somatic treatment. However, studies have shown it to be significantly lower in CKD children than in healthy children [11, 15, 16]. The results of our study in this area are consistent with the observations of other authors using the same PedsQL test [11, 16]. In contrast to other studies, we have found that the overall HRQoL depends on the particular method of treatment. A lower HRQoL was observed in children on HD when compared with patients treated conservatively or with PD. This confirms the validity of choosing PD as more beneficial to HD patients, also regarding HRQoL [12]. Similar observations were made by other authors [24]. It should be emphasised that most publications comparing the QoL based on the dialysis type and demonstrating the superiority of PD over HD concerned adults [25, 26]. They emphasised greater autonomy in PD patients and pointed out that HD limits the daily life activities to a greater extent [27–29]. Our study identified differences in perception of QoL components related to the treatment methods. Children treated with HD and PD reported lower physical and social functioning than CT patients. The specificity of dialysis treatment and changes in the body may be the source of physical discomfort and perceived limitations in daily activity. Moreover, ESRD reduces the possibility of participating in peers’ lives. Low social self-perception may result in less active participation in the society during adult life [30, 31]. Contrary to our expectations, the perception of emotional and school functioning of dialysis patients was not different from CT children. This suggests that the disease itself, regardless of the stage of severity, is a source of emotional problems. It is impossible to exclude the state of relative elevated perception of positive emotion among dialysis patients when compared with other patients with CKD. That state was described by other authors [16] and “response shift” theory is taken into consideration to explain it [32]. In the course of the disease, the increased participation of “defence mechanisms” in one’s psychical functioning cannot be excluded. This could prevent the emotions from becoming more negative over time.

Any possible differences pertaining to school are likely to be distorted as a result of changes in teaching methods. Our study showed that children treated with PD and HD took advantage of private tutoring considerably more often than CT children. Nonetheless, the evaluation of school functioning is generally low. This emphasises that the school achievements should be improved, the teaching programme should be adapted to the individual circumstances of the patients and any efforts of the pupils should be encouraged.

Our research demonstrated that the HRQoL in CKD children on CT is poorer than that of healthy children. To the best of our knowledge, thus far, only Gerson et al. [33] have evaluated the quality of life in a group of children with mild-to-moderate CKD. Our observations are similar in line with Gerson. They illustrate that the significant impairment of the perceived quality of life occurs at an early stage of CKD. Similar to Gerson’s research, our study found no differences based on the stage of CKD in the overall scale or in the PedsQL subscales. The most significant differences in regard to norms were found in school functioning, which is also consistent with Gerson’s observations. This may be caused, among other things, by metabolic disorders and the functions of the central nervous system in kidney disease, reported fatigue, sleep disorders or poor school attendance [33].

Our research revealed no influence on the perceived quality of life in the group of all children with CKD regardless of treatment methods, age, gender, the disease duration, place of residence or distance from a nephrology centre. Similar observations were made by Amr et al. while studying pre-dialysis and HD children [34]. They did not find any significant correlations between psychiatric disorders and age, gender or the duration of CKD. According to the authors, the quality of life is more conditional on encountered problems than demographic factors. However, we found that a professional activity of the mothers, in addition to those of the fathers, influenced positively the evaluation of physical functioning. It is possible that working mothers expect more self-independence and responsibility from their children. This results in a better estimation of the physical functioning.

In our research, it was observed that children with both parents reported better social functioning and experienced a higher QoL than children from single-parent families. Both parents can provide a greater sense of security, support and stability which determines higher self-esteem. This psychological factor in studies of adult pre-dialysis patients was indicated as a particularly important determinant of well-being [31]. We did not find any effect of the duration of nephrological care on HRQoL. This is consistent with the observations of other authors who demonstrated that although early nephrological care improves the clinical condition of CKD patients, it is not sufficient to minimize the psychosocial aspects resulting from kidney disease [6].

In our research, we also assessed the parents’ perception of their children’s quality of life. It varied dependant on the degree of CKD severity and intensity of health care. Main caregivers of CT patients evaluated their children’s overall functioning and within all subscales significantly higher than parents of dialysis patients. Main caregivers of HD children assessed their children’s QoL as the lowest. Perception of the children`s HRQoL by “second” parents was different than main caregivers (usually the mother). Probably, the “second” parents may have a lower threshold for detecting psychological distress in their children. A systematic review concluded that convergence of rating is better on the subscales related to directly observable functioning and with no emphasis on subjective qualities [35, 36].

The literature data regarding the agreement of the child self-report and parent/proxy reports are conflicting. In some studies, children evaluated themselves more negatively than their parents did and vice versa in other studies [34, 37]. Factors that influence these disparities are unclear. Our study showed that the perception of HRQoL did not differ between children on HD and their primary caregivers (low for both the child and the parents). In turn, significant differences were present in the assessment of the overall HRQoL and its components in all the groups of patients, as well as the CT (with exception of emotional functioning) and PD (with exception of school functioning) subgroups between children and their main caregivers. Parents rated their children’s quality of life lower than the children themselves. Such discrepancies between the parents and their children were also discussed by Morrow et al. [38] who studied children suffering from various chronic diseases. The above indicates that differences in perception of life in children and their parents should be included in psychological support programmes for families of CKD children. It is possible that, due to the shorter time perspective and their temporal orientation to the present, children view their life situation more positively than their parents that expect a uncertain and difficult future of their children. Worse perception of school and physical functioning among parents may adversely affect a child’s motivation for physical activity and studying and may have negative repercussions in the child’s further development. It can also be a source of conflicts between parents and their children that, according to Tong et al. [39], may result in negative consequences for children with health problems.

Our study has several limitations. The varying number of subjects in the individual groups of studied children may limit the statistical analysis. However, obtaining materials from larger groups would require international research with an inclusion of a specific cultural context. It should be noted that in comparison with the data in the literature, the groups tested by us are relatively large. Our results pertain to the Polish population. They may not be the representative of other populations; however, we believe that they contribute with important data concerning the consequences of CKD. Data were obtained from most parents; however, only some of them completed the PedsQL tests, particularly the few parents who are not the primary caregivers. The above may not only indicate the polarization of the tasks associated with caring for a sick child but also the actual emotional split within the family. This suggests the need for further research on social structure in families with children suffering from CKD and to determine the actual role of the individual family members. It is worth mentioning that the present study had a cross-sectional design which means that no conclusion can be drawn in regard to the causality of the observed relationships. We are also aware that numerous factors that may determine the quality of life (both somatic and related to the families’ socio-economic status) have not been taken into account. Due to the small number of HD children (currently, a small percentage of children with ESRD treated with haemodialysis), the observation that this group requires special psychological support is questionable.

In summary, the health-related quality of life in children with CKD is lower than in healthy children, already in the early stages of the disease. The lack of significant differences in the perception of emotional functioning depending on the stage of CKD indicates the influence of the disease itself on the child’s mental state. This suggests the necessity of psychological help for children with CKD practically immediately after the diagnosis. Mainly, support should be given in the areas of school education and emotional functioning. Children on HD require special support on account of the lowest demonstrated overall HRQoL. Children’s lower rating of the quality of life observed by their parents may render the patients unmotivated and adversely affect their adaptation to life in later years. It also may create conflicts between the parents and their children. Further multi-centre research should be conducted in order to develop viable programmes for improving the HRQoL.
